# Enhanced Feedstock Processability for the Indirect Additive Manufacturing of Metals by Material Extrusion through Ethylene–Propylene Copolymer Modification

**DOI:** 10.3390/polym16182658

**Published:** 2024-09-20

**Authors:** Thomas Forstner, Simon Cholewa, Dietmar Drummer

**Affiliations:** 1 Institute of Polymer Technology, Friedrich-Alexander-Universität Erlangen-Nürnberg (FAU), Am Weichselgarten 10, 91058 Erlangen, Germany; 2 Collaborative Research Center—Additive Manufacturing (CRC 814), Friedrich-Alexander-Universität Erlangen-Nürnberg, Am Weichselgarten 10, 91058 Erlangen, Germany

**Keywords:** material extrusion, ethylene–propylene copolymer, feedstock, tool steel, filament

## Abstract

Filament-based material extrusion (MEX) represents one of the most commonly used additive manufacturing techniques for polymer materials. In a special variation of this process, highly filled polymer filaments are used to create metal parts via a multi-step process. The challenges associated with creating a dense final part are versatile due to the different and partly contrary requirements of the individual processing steps. Especially for processing in MEX, the compound must show sufficiently low viscosity, which is often achieved by the addition of wax. However, wax addition also leads to a significant reduction in ductility. This can cause filaments to break, which leads to failure of the MEX process. Therefore, the present study investigates the influence of different ethylene–propylene copolymers (EPCs) with varying ethylene contents as a ductility-enhancing component within the feedstock to improve filament processing behavior. The resulting feedstock materials are evaluated regarding their mechanical, thermal and debinding behavior. In addition, the processability in MEX is assessed. This study shows that a rising ethylene content within the EPC leads to a higher ductility and an enhanced filament flexibility while also influencing the crystallization behavior of the feedstock. For the MEX process, an ethylene fraction of 12% within the EPC was found to be the optimum regarding processability for the highly filled filaments in MEX and the additional processing steps to create sintered metal parts.

## 1. Introduction

**Figure 1:**
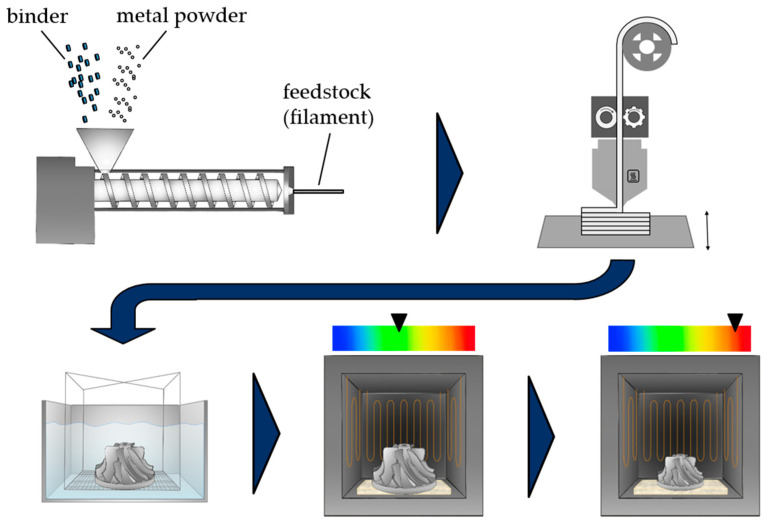
Typical processing scheme for indirect additive manufacturing of metal parts via filament-based MEX-MSt/M.

Filament-based material extrusion (MEX) is a widely used and easily accessible additive manufacturing technology that has experienced major market growth over the last decade [1]. MEX is defined as the extrusion of material through an orifice or a nozzle to create a three-dimensional component in a stepwise process according to DIN EN ISO/ASTM 52900 [2]. In addition to the processing of neat, commodity, and engineering polymer materials like polylactic acid (PLA) or acrylonitrile butadiene styrene (ABS), which are the most popular materials for MEX [1], the technology also allows for the processing of high-performance thermoplastic polymers like polyether ether ketone (PEEK) [3] or filled polymer systems [4,5]. In this regard, the incorporation of filler particles or fibers can be used to target directional mechanical properties [4] or integrate additional functionalities into additively manufactured parts and, therefore, improve heat transfer [6], electrical conductivity [7] or create electromagnetic interference-shielding properties [8]. A special process variant of MEX, which is also based on the processing of highly filled polymers, can be used to produce metal parts indirectly and is declared as the filament-based additive manufacturing of metals by material extrusion in a multi-step process (MEX-MSt/M, as defined in [2]). The process is typically composed of multiple steps to create the final metal part, as depicted in Figure 1. It involves the additional stages of debinding and sintering after the initial shaping process of MEX [9]. These steps also resemble the well-established metal injection molding (MIM) process [10]. While this special injection molding technique enables an efficient production of especially small and complex metal parts in high quantities [10], MEX aims at lower quantities while further enhancing the freedom of design for metal parts. However, both processing methods can achieve densities and mechanical properties similar to conventionally processed metal parts after sintering [11,12].

Although integrating fillers into filament feedstocks opens up a variety of new fields of applications [13], the processing of filled or even highly filled polymers comes with multiple challenges that need to be overcome to fully unfold their potential within MEX. One of the main requirements for the processing of highly filled feedstocks via MEX- MSt/M, amongst many other general demands for the indirect manufacturing of metals [14], is a sufficiently low viscosity and a good interaction of particles and matrix [15]. Low viscosity values are often achieved using low-molecular components like waxes [16,17]. Although the use of waxes improves melt flowability and can enhance particle wetting for a more homogeneous distribution of particles, higher amounts of waxes also lead to a significant change in mechanical behavior [18]. This manifests in less ductile material behavior, which can cause difficulties in spooling and feeding of the filament and can ultimately lead to filament breakage, which may result in a failure of the MEX process. 

One already patented approach by BASF to maintain the filament flexibility at high filler loadings is the establishment of a core–shell filament structure, in which the filament-shell acts as a flexible supporting material for the highly filled core material [19]. Another way to improve filament flexibility for MEX-MSt/M is the introduction of ductility-enhancing components like elastomers [20] or thermoplastic elastomers (TPEs) [11] into the feedstock. Ethylene–propylene copolymers (EPCs) are an example for thermoplastic olefin elastomers (TPOs), a sub-category of the TPEs. They are often used in combination with other polyolefines like polypropylene (PP) to increase their low-temperature impact toughness in an automotive industry [21]. EPCs, like other TPOs, are typically characterized by highly ductile material behavior and softness [22], which can be tailored through the ratio between ethylene and propylene segments. This ratio can also be expressed by the ethylene content (C2 content), whereas a higher C2 content, for example, leads to reduced stiffness [21,23] and higher impact strength [21] as a sole copolymer [23] as well as in high impact PPs [21]. Although TPEs are already employed within the existing literature for processing through MEX [24], there is no systematic research carried out on the impact of different EPCs on filament and processing properties in MEX-MSt/M. 

Therefore, this study aims to create a better understanding of the influences of EPC modification on the filament properties and processing behavior in MEX. This will be achieved through a targeted variation in EPCs in combination with two different binder components in order to widen the choice of materials for the indirect manufacturing of metals.

## 2. Experimental Section

### 2.1. Materials

In the present study, a commercially available low-density polyethylene (PE-LD) with an MFR of 65 g/10 min and polyethylene wax (PE wax) “E25K” by Deurex (Elsteraue, Germany) were used as base-binder components for the metal feedstock. Three different commercially available ethylene–propylene copolymers were employed as ductility-enhancing components. EPCs differ in terms of their ethylene content, as well as in viscosity. The ethylene contents were varied in steps of 6 wt. %, 12 wt. %, and 15 wt. %. The binder fraction was kept constant at 40 vol. % of the compound and was either composed of PE-LD or PE wax as a base in combination with an EPC fraction or is solely composed of EPC. As a filler, a tool steel powder of 1.2343 (H11) by m4p (Magdeburg, Germany) was used within the feedstock at a constant share of 60 vol. %, which is a regular filler content for MSt/M [9,25] or MIM [14] and corresponds to 92.8–93.0 wt. % depending on the density of the binder composition. 

**Figure 2:**
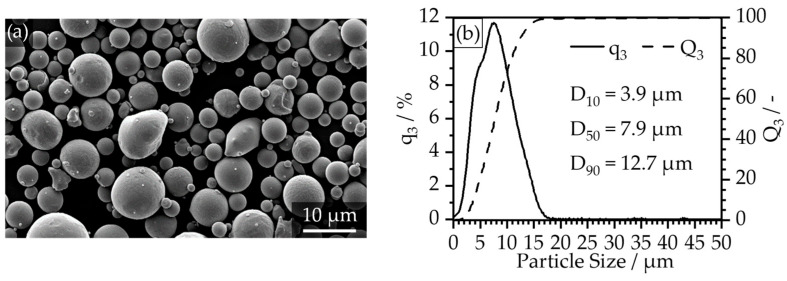
Particle characteristics of the 1.2343 (H11) tool steel powder: (**a**) SEM image of the spherical particles; (**b**) volumetric particle size distribution determined by dynamic picture analysis on a Camsizer X2 by Mictrotrac Retsch (Haan, Germany).

The filler particles can be characterized as primarily spherical with a small fraction of irregularly shaped particles, which is demonstrated by Figure 2a. The particle size distribution is typical for MIM and extrusion-based indirect additive manufacturing techniques with a D_10_ = 3.9 µm, a D_10_ = 7.9 µm and a D_90_ = 12.7 µm. Furthermore, the detailed feedstock compositions are depicted in Table 1, where components 1 and 2 build the binder system with a combined share of 40 vol. %.

### 2.2. Processing

The materials were compounded prior to filament extrusion. Therefore, the different binder materials were dry-blended in a tumbling mixer and dried at 60 °C for 12 h before compounding. For the compounding step, a co-rotating twin-screw extruder ZSE HP17 by Leistritz (Nuremberg, Germany) was employed at a constant screw speed of 100 rpm and temperatures from 140 °C at the hopper to 180 °C at the nozzle. The strands were cooled down on a cooling plate and pelletized afterward. The granular material was then used to produce tensile bars in MIM and to prepare filaments for MEX. 

For mechanical evaluation of the green-part properties, dog bone tensile bars with a cross-section of 10 mm × 2 mm within the parallel testing area were manufactured employing the MIM technique [10] on an Ergotech 25–80 injection molding machine by Sumitomo Demag (Schwaig, Germany) within a dual cavity mold. The green parts were produced with a constant mass temperature of 200 °C and a mold temperature of 45 °C. The injecting speed was kept constant at 30 mm/s, which resulted in a total cycle time of 50 s. 

For further processing via MEX and for the evaluation of the filament flexibility via determination of the minimum bending radius, filaments with a round diameter of 1.75 mm were extruded at a mass temperature of 200 °C using Composer 350 by 3devo (Utrecht, The Netherlands). After extrusion, the filaments were spooled onto a standard FFF spool with an inner diameter of 120 mm, if possible. 

Furthermore, the processability in MEX was tested using a Funmat HT by Intamsys (Shanghai, China) equipped with a 0.8 mm nozzle. The MEX machine was upgraded with a dual direct drive extruder by BondTech (Värnamo, Sweden). The general extruding ability of the filaments was tested by feeding 50 cm excerpts of the filaments from the top of the opened building chamber through the feeding mechanism to extrude material at temperatures of 220 °C and 240 °C, if possible. Square specimens with an edge length of 20 mm and a height of 4 mm were then manufactured at a 20 mm/s printing speed at 220 °C and 240 °C nozzle temperatures for the material combinations with 12% ethylene within the EPC fraction and PE-LD or PE wax as a base binder. 

**Figure 3:**
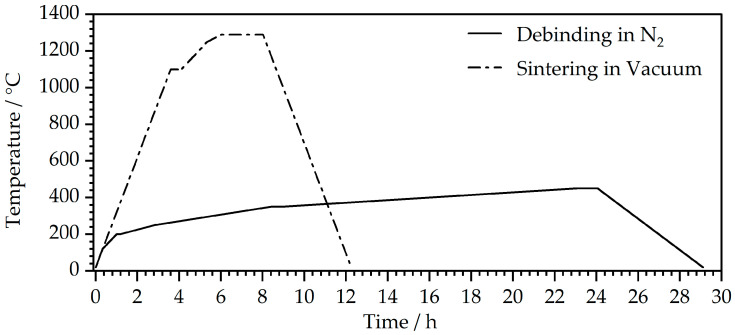
Temperature profiles used for thermal debinding and sintering of the MEX samples.

Afterward, the square samples were placed in n-heptane at 60 °C for 24 h for solvent debinding. The remaining polymer fraction was then thermally removed in a second debinding step using a GLO 40/11 debinding furnace by Gero (Neuhausen, Germany), before being sintered in an HTK 25 sintering furnace by Gero (Neuhausen, Germany) to obtain compact metal parts. Thermal debinding was carried out under a constant flow of nitrogen and sintering at a vacuum of 5 *×* 10^−3^ mbar to prevent the metal material from oxidizing. These steps were conducted by employing the temperature profiles depicted in Figure 3.

### 2.3. Methods

The feedstocks’ thermal processing and decomposition behaviors were assessed using differential scanning calorimetry (DSC) and thermogravimetric analysis (TGA). DSC samples weighed approximately 10 mg, while TGA specimens weighed around 20 mg. Both samples were obtained from the filament material after extrusion to ensure a similar thermal history close to the MEX process. The DSC measurements were carried out on a Discovery DSC 2500 by TA Instruments (New Castle, DE, USA) using nitrogen as a purging gas. The measurement protocol included two heating cycles from −40 °C to 180 °C with one cooling cycle in between, at heating and cooling rates of 10 K/min. TGA measurements were conducted using TGA Q5000 from TA-Instruments (New Castle, DE, USA) with a constant heating rate of 10 K/min. The measurement was performed under a continuous nitrogen purge up to a maximum temperature of 700 °C. 

Mechanical testing for the injection-molded specimens was conducted in accordance with DIN EN ISO 527-1 [26] using a 5948 MicroTester by Instron (Norwood, MA, USA), equipped with an optical extensometer. The testing speed was set at 1 mm/min and at 0.33 mm/min for determining Young’s modulus. Additionally, filament flexibility was evaluated by testing the minimum bending radius according to VDI 3405 [27]. Therefore, two different 1 m long strand sections of the extruded filaments were fed through a bending template with stepped radii. To assess powder binder adhesion and breaking morphology after tensile testing, scanning electron microscope (SEM) was performed, employing a Gemini Ultra-Plus by Carl Zeiss (Oberkochen, Germany). The gold-sputtered samples were examined with a secondary electron detector at a magnification of 2.000× and an acceleration voltage of 10 kV. The soluble binder fraction was determined for the MIM-fabricated samples by weighing the specimens before and after solvent debinding in n-heptane at 60 °C for 24 h and subsequent drying at 40 °C for 12 h on a 390HA-125SM precision scale by Precisa Gravimetrics (Dietikon, Switzerland). 

## 3. Results and Discussion

### 3.1. Thermal Behavior

**Figure 4:**
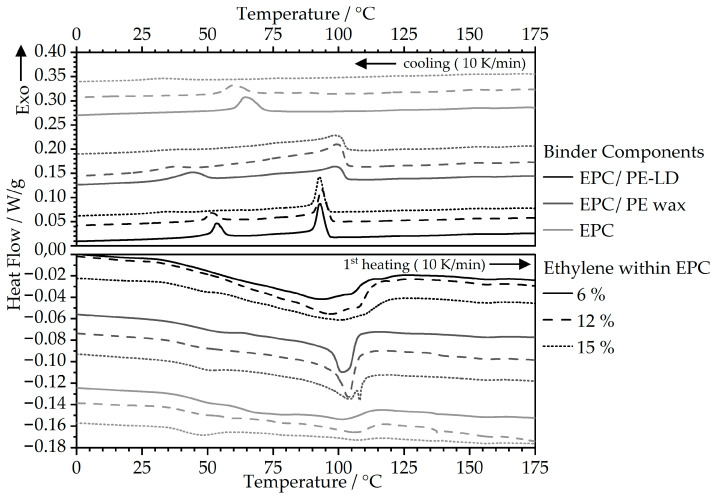
DSC measurements of the highly filled feedstocks for the evaluation of melting and crystallization behavior.

The DSC measurements were conducted to evaluate thermal behavior in terms of melting and crystallization behavior in MEX. Therefore, the thermograms for the first heating and cooling rates are shown in Figure 4. The heating curves show distinct differences concerning the binder composition. While the pure EPC binders are characterized by a very little pronounced peak with gradual melting of the material, the combined binder systems with parts of PE-LD and PE wax display clear melting areas due to their higher crystallinity compared to the mainly amorphous EPC materials. Here, the feedstocks with PE wax/EPC binders show a narrow and sharp melting peak area, whereas the PE-LD/EPC binders are characterized by a broader melting peak area. This behavior is also independent of the EPC type used. 

The crystallization behavior of the materials, which is particularly important for the consolidation in MEX, can be described based on the peaks of the cooling curves. In this regard, the neat EPC matrices show clear crystallization peaks within relatively low temperature ranges, from 30 °C to 70 °C, with a shift in the crystallization onset and peak temperatures towards lower temperatures combined with a reduction in peak height for a higher fraction of ethylene within the copolymer. The shift towards lower temperatures can be attributed to a hindered crystallization process when a higher amount of ethylene is present within the polymer chains. Therefore, the ethylene segments act as defects within the macromolecules [28] that are slowing down the crystallization process [23], leading to a higher defect concentration within the non-crystallized areas [28] and an overall reduction in crystallinity [23]. Especially at a higher C2 content of 15 wt. %, the crystallization process cannot even take place at higher melt undercooling, as demonstrated in Figure 4, resulting in an almost complete distinction of the EPC crystallization peak for the feedstocks containing the EPC with an ethylene fraction of 15 wt. %. This behavior can also be observed when the EPC is combined with PE-LD or PE wax within the binder fraction. Furthermore, the presence of the short-chained wax segments within the binder leads to higher crystallization onset temperatures compared to PE-LD, as reported in similar studies [18].

### 3.2. Mechanical Properties

#### 3.2.1. Tensile Properties

**Figure 5:**
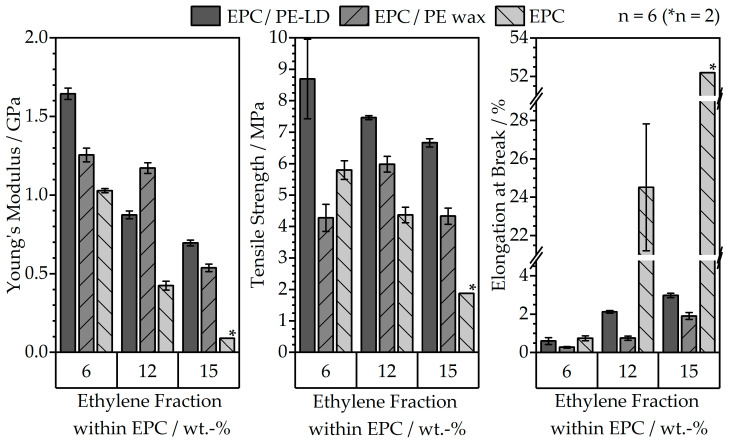
Mechanical values for different feedstock compositions and ethylene fractions within the EPC, determined by tensile testing of the MIM-fabricated dog bone specimen.

The mechanical properties of the MIM-fabricated green parts were evaluated in tensile testing and are represented in Figure 5. Regarding the mechanical properties, a rising ethylene fraction within the EPC primarily leads to a substantial increase in ductility and reduction in the stiffness of the material, which are reflected by the decrease in Young’s modulus and the high elongation at break of up to 52% for a neat EPC matrix with 15 wt. % ethylene. This behavior is particularly evident when observing the feedstocks with the neat EPC matrices, which achieve the highest values for elongation at break and the lowest moduli, but also transfer to the multi-component binder systems. 

Concerning the multi-component binders, the introduction of PE wax leads to a significant decrease in elongation at break compared to the neat EPC and the PE-LD/EPC binders to total values for the different ethylene fractions within an EPC of under 2% elongation. When combining the EPC materials with PE-LD, a maximum elongation at break of 3% can be achieved. Overall, the least ductile material combination is achieved by combining a low ethylene fraction of 6 wt. % within the EPC and PE wax. 

#### 3.2.2. Filament Flexibility

**Figure 6:**
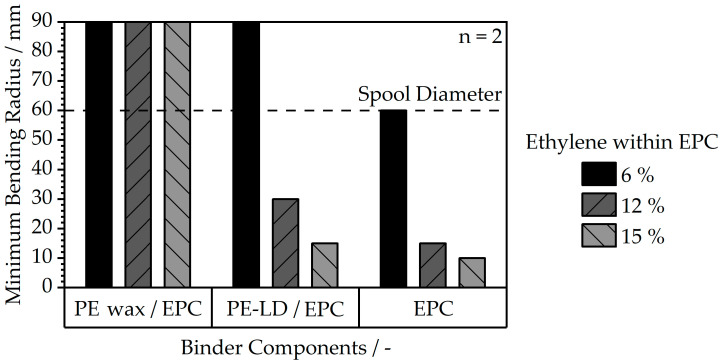
Minimum bending radii for 1.75 mm filaments determined according to VDI 3405 [27] for different feedstock compositions and ethylene fractions within the EPC.

The values for filament flexibility are depicted in Figure 6 by the minimum bending radii of the filaments, and they resemble the trends observed in tensile testing. 

Here, the lowest ethylene fraction within the EPC also leads to the least flexible filament. In addition, the lowest minimum bending radii, which indicate a highly flexible filament, are achieved at highethylene fractions within the EPC. In general, the filaments with a sole EPC binder demonstrated the highest flexibility and ductility; as a result, winding and unwinding on standard FFF spools were possible to ensure a continuous supply of filament. The filaments with a PE wax portion, however, experienced the least favorable properties with high minimum bending radii for spooling and, therefore, would break easily due to their highly brittle behavior, as already indicated by the tensile properties in Figure 5. As a result, for the processing trials, it was only possible to use filament segments of the materials with minimum bending radii of more than 60 mm for fabrication of specimens in MEX. The binder systems without wax, especially medium- and high-ethylene fractions within EPC, result in a low minimum bending radius and ensure continuous filament winding.

#### 3.2.3. Fracture Morphology

**Figure 7:**
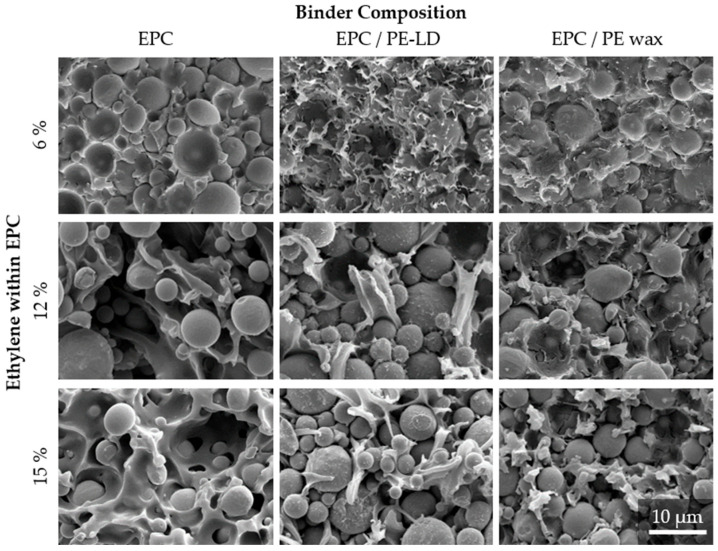
SEM images of the fracture surfaces of the tensile bars after tensile testing at 2.000× magnification for the evaluation of fracture morphology and particle–matrix interactions.

The fracture surfaces of the specimens after tensile testing, depicted in Figure 7, confirm the corresponding mechanical performance of the material on a microscopic scale. The micro-morphology shows a clear tendency towards more ductile breaking behavior in the case of materials with a pure EPC binder from an ethylene content of 12% and upwards in the EPC, which can be observed. This behavior is displayed by an increasing fibrillation of the matrix between the particles with a rising C2 content. 

On the contrary, particle wetting for a neat EPC matrix is very poor regardless of the ethylene content in the EPC. This is indicated by an exposed particle surface and cratering around the particles, which is typical for poor particle–matrix-interactions. This behavior is maintained in combination with PE-LD, although particle wetting improves here. On the other hand, the feedstocks with wax and EPCs show a very good particle–matrix bond, especially with a low ethylene content in the EPC. Here, the particles are covered by the matrix, showing good wetting behavior, which is also favorable for a homogeneous particle distribution within highly filled polymers and reduces the probability of defects like pores or cracks in the final part as a result of segregations in the context of the indirect manufacturing of metal parts [29]. Although the particle wetting behavior improves with the addition of wax, more brittle breaking behavior is evident for the wax-containing feedstocks, which reflects the results from the mechanical characterizations.

### 3.3. Debinding Behavior

#### 3.3.1. Solvent Debinding

**Figure 8:**
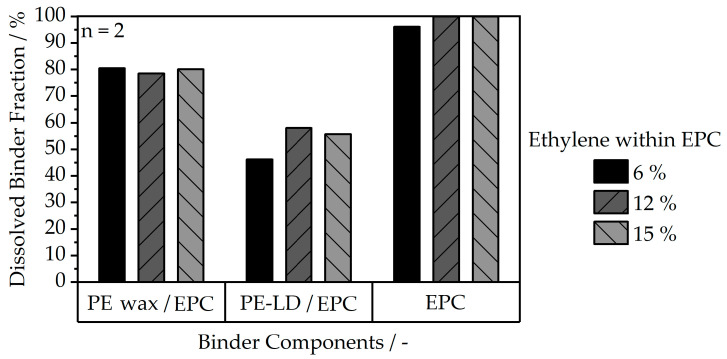
Dissolved binder fraction for the different feedstock compositions after solvent debinding of dog bone specimens in n-heptane at 60 °C for 24 h.

For the creation of a defect-free final part, partial removement of the binder fraction through a solvent debinding step prior to thermal debinding is crucial. Thereby, open pore channels are created, and free evaporation of the remaining polymer in thermal debinding is enabled [10]. In this context, all material combinations within the present study demonstrate sufficiently good solubility, as depicted in Figure 8. 

Besides EPC, the co-binder type poses the primary influence on the solvent debinding behavior, while the amount of ethylene within EPC only has a minor impact. Furthermore, the neat EPC systems are almost entirely dissolved, which leads to a loss of the specimen geometry. As a result, EPC cannot be used as a single-component binder system and should always be accompanied by an insoluble or partially soluble co-binder as a backbone. When combining EPC with PE-LD, the soluble fraction was the lowest among the investigated materials, with values between 46.2% and 58 %. However, these values can still be considered sufficiently high for creating dense parts, compared to values from the literature of 41.1% for producing defect-free parts [30]. The values indicate that only a small fraction of PE-LD is being dissolved when considering the total ratio of both components within the compounds. On the contrary, the PE wax co-binder is partially soluble, leading to an increased overall dissolved binder fraction of 78.5% to 80.47%, which creates an enhanced open-porous network between the filler particles, and, therefore, enables beneficial degassing throughout the thermal debinding step.

#### 3.3.2. Thermal Debinding

**Figure 9:**
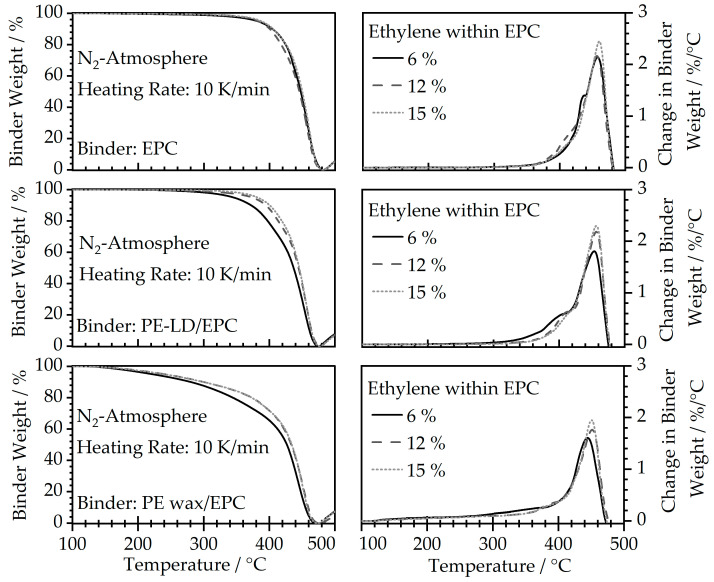
Thermal decomposition behavior of feedstocks determined by TGA measurements in nitrogen atmosphere.

The thermal degradation behavior of the binder fraction within the feedstocks was determined by TGA measurements and is depicted by the graphs in Figure 9. The measurements were performed while constantly purging with nitrogen to replicate the actual debinding process as accurately as possible, which is usually carried out in a vacuum or in an inert gas atmosphere for most steels and steel alloys [31,32]. Although the nitrogen purge should prevent the steel from oxidization, an increase in weight can be observed after the complete binder decomposition at temperatures of around 480 °C and above for all samples, due to a potential incomplete exclusion of oxygen. However, it also has to be noted that the present measurements were carried out at a heating rate of 10 K/min, which is significantly faster than the real thermal debinding process, which leads to a shift in the degradation peak towards higher temperatures as a result of the inertia of the system. Concerning the degradation behavior of the different compositions, a lower ethylene fraction within EPC leads to a slight broadening of the decomposition peak towards lower temperatures, compared to a more rapid degradation behavior for a higher ethylene fraction. However, this behavior can mostly be neglected when applying a two-step debinding process, as EPC poses as the main soluble portion within the feedstock and should be removed at the time of thermal debinding. The PE-LD/EPC feedstocks show no noticeable differences in decomposition behavior compared to the neat EPC materials. Both are characterized by relatively rapid degradation behavior, which requires lower heating rates in thermal debinding compared to the PE wax/EPC materials. Here, the wax addition leads to a slow and gradual decomposition of the binder, which is favorable for avoiding gas accumulation within the part and possible crack formation.

### 3.4. Processability in MEX

**Figure 10:**
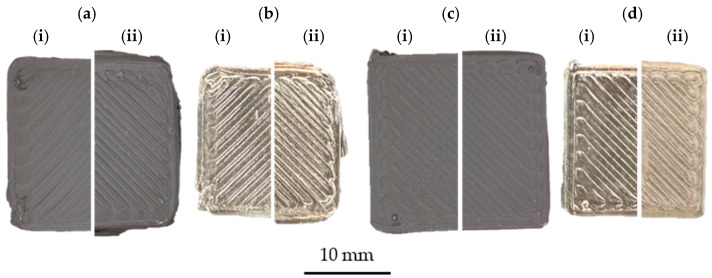
MEX-printed samples in their green (**a**,**c**) and sintered (**b**,**d**) state for 220 °C (**i**) and 240 °C (**ii**) nozzle temperatures based on feedstocks with 12 wt. % ethylene within EPC, PE wax/EPC matrix (**a**,**b**) and PE-LD/EPC matrix (**c**,**d**).

The general extrudability at 220 °C and 240 °C was demonstrated for the materials containing PE-LD and those with PE wax and a C2 content of at least 12 wt. %. The low hardness, in combination with the very high flexibility of the neat EPC filaments, prevents homogeneous feeding and favors buckling, a common reason for the process failure of filament-based MEX [33], of the filament in the feed towards the hot end, which prevents sufficient pressure build-up for extrusion. As a result, the processing for these materials was not possible. On the contrary, the feedstocks with PE wax or PE-LD in combination with an ethylene content of 12 wt. % within the EPC showed excellent processability, and the fabrication of squared samples was possible for both temperatures. In this regard, Figure 10 displays the successfully fabricated green and sintered parts for the different temperatures and material combinations. 

The use of PE-LD as a co-binder enabled good dimensional stability throughout manufacturing (Figure 10c) at the investigated parameter sets. At the same time, the PE wax/EPC combination caused blurring of the square outline due to the melt viscosity being too low, at a temperature of 240 °C. However, this behavior can be reduced by lowering the die temperature to 220 °C, as shown in Figure 10(ai). Furthermore, the samples retained their shapes after sintering (Figure 10b,d) with a standard shrinkage for MEX-MSt/M [33], but also showed cracks for the feedstock containing PE-LD within their cross-section. The sample with a PE wax binder fraction could be obtained as dense and crack-free. This can be deduced from the higher amount of binder removed after solvent debinding.

## 4. Conclusions

The present study evaluated the influence of adding different EPC types, characterized by a varying amount of ethylene within the copolymer, as a processing modifier within the context of MEX-MSt/M. The thermal behavior was found to be significantly influenced by the amount of ethylene within EPC, delaying the crystallization process and leading to a reduced overall crystallinity at higher C2 contents. Regarding the mechanical properties and filament flexibility, an increase in ethylene content within EPC leads to higher ductility but lower stiffness, and, therefore, a more flexible filament. PE wax, on the other hand, reduces elongation at break, making the material more brittle and creating a less flexible filament, while PE-LD offers moderate flexibility, resulting in sufficient flexibility for filament winding. Concerning solvent debinding, neat EPC binders dissolve too much during solvent debinding, leading to shape loss, whereas combinations with PE-LD or PE wax show better performance. PE wax enhances open porosity, aiding in beneficial degassing during the thermal debinding step. For thermal debinding, EPCs with higher ethylene content degrade rapidly compared to a lower C2 content. Furthermore, the addition of PE wax broadens the degradation peak, reducing the risk of gas accumulation and cracking, compared to rapid degradation for the sole EPC and PE-LD/EPC binders. Finally, the processability in MEX-MSt/M was successfully proven for the binder compositions with a C2 content of 12 wt. % and PE wax or PE-LD, forming stable parts. Even though sintered parts could be obtained for both material combinations, cracks within the parts based on the PE-LD/EPC feedstock were present.

This study found that the choice of binder composition significantly impacts the thermal, mechanical, and processing outcomes in MEX. Future research in this context may focus on further enhancing the sintered properties by targeting the processing strategy in MEX and the follow-up processes to avoid crack formation. 

## Figures and Tables

**Table 1:** Feedstock compositions used within this study.

Component 1		Component 2 (EPC)		Metal Powder
Type	Share /vol. %	EPC Share /vol. %	Ethylene in EPC ^1^ /wt. %	Share /vol. %
-	-	40	6	60
PE-LD	20	20	6	60
PE wax	20	20	6	60
-	-	40	12	60
PE-LD	20	20	12	60
PE wax	20	20	12	60
-	-	40	15	60
PE-LD	20	20	15	60
PE wax	20	20	15	60

^1^ According to the material data sheets and material test certificates of the supplier.

## Data Availability

The data presented in this study are available upon request from the corresponding author.
